# Genome-Wide Identification of *Juglans regia* GABA Transcription Factors and Expression Pattern Analysis in Response to Abiotic Stress

**DOI:** 10.3390/genes16111290

**Published:** 2025-10-30

**Authors:** Yulian Wang, Bin Wang, Wei Chen, Bin Wang, Tianlei Li, Xiang Luo, Jia Xue, Xinyi Wang, Jing He, Xiujuan Wang

**Affiliations:** 1 College of Forestry, Gansu Agricultural University, Lanzhou 730070, China; 18215124980@163.com (Y.W.);; 2 Institute of Fruits and Vegetables, Xinjiang Academy of Agricultural Sciences, Urumqi 830091, China; wangbin@xaas.ac.cn (B.W.);; 3 Wolfberry Harmless Cultivation Engineering Research Center of Gansu Province, Lanzhou 730070, China

**Keywords:** γ-aminobutyric acid, salt stress, drought stress, metabolic pathway, phytohormone regulation

## Abstract

**Background/Objectives**: γ-aminobutyric acid (GABA), a non-protein amino, is synthesized from glutamic acid through the catalytic activity of glutamate decarboxylase (GAD). As a key signaling molecule, GABA plays a vital role in plant responses to abiotic stresses. To explore the potential involvement of the GABA gene family in *Juglans regia*’s response to environmental stressors, a comprehensive genome-wide identification and analysis of GABA-related genes was performed. **Methods**: The study examined their protein features, evolutionary relationships, chromosomal locations, and promoter cis-regulatory elements. Additionally, the expression patterns of GABA family genes were analyzed in *J. regia* seedlings subjected to salt and drought stress. **Results**: Genome analysis identified three main components of the GABA metabolic pathway in *J. regia*: glutamate decarboxylases (*GADs*), GABA transaminases (*GABA-Ts*), and succinic semialdehyde dehydrogenases (*SSADHs*). These genes were unevenly distributed across 14 chromosomes, with chromosome 10 containing the highest number. Promoter analysis revealed that about 80% of cis-acting elements were linked to plant hormone regulation, such as abscisic acid (ABA), and stress responses, including drought and high-salinity. Phylogenetic analysis showed that *JrGAD1* was distantly related to other *JrGAD* members, while certain *JrGABA-T* and *JrSSADH* genes formed closely related pairs. Under salt and drought stress, *JrSSADH23* expression was highly upregulated (2.60-fold and 2.24-fold, respectively), a trend observed for most *JrSSADH* genes. **Conclusions**: These findings offer valuable insights into the molecular basis of GABA metabolism in *J. regia*’s stress adaptation and identify promising genetic targets for developing stress-tolerant varieties.

## 1. Introduction

Abiotic stresses, including drought, salinity, and cold, are crucial environmental factors that impede plant growth and limit agricultural yields [1]. Upon encountering abiotic stress, plants often accumulate considerable levels of detrimental compounds, including reactive oxygen species (ROS), malondialdehyde (MDA), and methylglyoxal (MGO). These substances can damage cell membrane structure, disrupt physiological functions, and cause lipid peroxidation, ultimately leading to cell death [2,3]. Concurrently, plants activate intrinsic defense mechanism by upregulating stress-related genes to mitigate cellular damage. Key gene families involved in these resistance mechanisms, including *NHX1*, *SOS1*, *ERF*, those involved in the γ-aminobutyric acid (GABA) pathway (e.g., *GABA-T*, *GAD*, *SSADH*), and *LEA*, have been identified, and their molecular roles in abiotic stress responses are under extensive investigation [4,5,6].

GABA, a four-carbon amino acid, is not incorporated into proteins [7]. It was first identified in potato tubers in 1949 [8]. Subsequent research has revealed that GABA acts as a signaling molecule and participates in regulation of plant growth, development, and stress responses [9,10,11]. In plants, GABA metabolism occurs primarily via the GABA shunt mechanism [12,13]. This pathway starts with glutamate in the mitochondria, where glutamate decarboxylase (GAD) catalyzes the conversion of glutamate to GABA. GABA is then transaminated by GABA aminotransferase (GABA-T) to produce succinic semialdehyde (SSA). In the final step, succinic semialdehyde dehydrogenase (SSADH) converts SSA into succinate, which can enter the tricarboxylic acid (TCA) cycle or be transported to the cytoplasm [14]. The key enzymes involved in the GABA metabolic pathway include GAD, GABA-T, and SSADH. Among these, GAD is particularly notable for its high sensitivity to abiotic stress. Its gene expression and enzymatic activity are closely linked to GABA-mediated enhancement of plant stress tolerance [15,16]. For instance, in species such as *Nicotiana tabacum*, *Panax ginseng*, and *Triticum aestivum*, the expression of GAD gene is significantly upregulated under salt stress, leading to GABA accumulation and improved salt resistance [17,18,19]. However, the homeostasis of GABA in plant tissues is not governed solely by its biosynthesis; the rate of degradation also plays a critical role [7,20]. Studies on *Arabidopsis thaliana* mutants such as the GABA transaminase knockout mutant (*pop2-1*) and the T-DNA insertion mutant (*pop2-3*) have shown that under stress conditions, these mutants exhibit marked reductions in GABA levels, as well as in GABA-T and GAD activities [21], resulting in diminished salt tolerance. GABA-T contributes to plant adaptation under saline conditions by regulating nitrogen assimilation and carbohydrate distribution, thereby linking nitrogen and carbon metabolism in the roots [22]. SSADH is a mitochondrial enzyme [23]. Mutants defective in SSADH accumulate excessive ROS under light stress and temperature stress, leading to retarded growth and leaf damage [24]. Studies of *SSADH* gene mutations in *A. thaliana* have demonstrated that loss of SSADH function causes accumulation of reactive oxygen intermediates (ROIs), impaired plant growth, and increased sensitivity to environmental stresses [23].

Furthermore, exogenous GABA application can induce coordinated accumulation of endogenous GABA, proline, and total phenols, thereby enhancing the plant’s antioxidant defense system and mitigating salt-induced damage [25]. In pepper, treatment with exogenous GABA improves water uptake and germination rate under salt stress by modulating osmotic balance and maintaining membrane integrity. It also reduces MDA content, activates the endogenous GABA shunt, and enhances metabolic and antioxidant activity, collectively improving seedling growth [26]. Similarly, in *Nigella sativa*, exogenous GABA application significantly increases chlorophyll content, soluble sugars, proline, and catalase activity, which promotes growth and yield under drought stress [27].

As a major woody oil-bearing tree species, *Juglans regia* is extensively cultivated in Xinjiang and other regions of China [28]. The walnut industry is of significant economic importance, but its sustainable development is frequently threatened by abiotic stresses prevalent in these cultivation areas. However, its growth is often affected by abiotic stresses such as drought, saline-alkali and low temperature, resulting in decreased yield and quality [29]. Furthermore, as a perennial deep-rooted tree, *J. regia* cannot escape environmental stresses like annual crops, making its inherent stress resistance mechanisms particularly crucial for survival and productivity. Over long periods of evolution, plants have developed sophisticated survival strategies to cope with abiotic stresses. These mechanisms are mediated by the perception of stress signals, leading to the activation of stress-responsive genes and the initiation of downstream defense pathways [30]. GABA functions as an essential signaling and metabolic agent that mediates plant adaptive responses to environmental stresses. Through its involvement in multiple metabolic and signaling pathways, GABA contributes substantially to enhancing plant tolerance to diverse abiotic stressors [12,31]. In recent years, members of the GABA-related gene family have been identified in *Gossypium hirsutum* [32], *Malus domestica* [33] and *Populus trichocarpa* [34]. However, the genomic organization and expression profiles of the GABA gene family in *J. regia* remain largely uncharacterized. Considering the economic importance of *J. regia* and the severe impact of abiotic stresses on its cultivation, along with the research gap on the GABA-mediated stress response pathway in this species, this study employed bioinformatics approaches to systematically identify GABA-associated genes in the *J. regia* genome. Furthermore, the expression patterns under drought and salt stress, with the goal of elucidating the molecular mechanisms of stress resistance in *J. regia* and providing candidate genes for breeding stress-tolerant *J. regia* varieties were analyzed.

## 2. Materials and Methods

### 2.1. Materials

The experimental material consisted of Xinjiang wild walnut (*J. regia*) seedlings, grown in a greenhouse at the Institute of Fruits and Vegetables, Xinjiang Academy of Agricultural Sciences. The greenhouse was maintained under the following conditions: an average daily temperature of 25 ± 3 °C, a nighttime temperature of 20 ± 3 °C, and a relative humidity of 60 ± 5%. After germination in January 2024, the seeds were sown in pots (25 cm in height, 17 cm inner diameter) containing a soil–peat mixture at a 2:1 ratio. During cultivation, seedlings were irrigated weekly with tap water and fertilized every 15 days with Hoagland’s nutrient solution (Beijing Coolaber Technology Co., Ltd., Beijing, China). Seedlings that developed five compound leaves and displayed uniform growth were selected for subsequent experiments.

### 2.2. Identification and Analysis of J. regia GABA Branch Gene Family Members

Genomic data for *J. regias* (GCF_001411555.2) and its annotation were sourced from NCBI (ncbi.nlm.nih.gov, accessed on 6 May 2025). Protein sequences for the GAD, GABA-T, and SSADH families, key components of the *A. thaliana* GABA metabolic pathway, were retrieved from the *A. thaliana* database (https://www.arabidopsis.org/, accessed on 6 May 2025) [35]. These sequences served as the foundation for further investigation. The sequences comprised six *GAD* genes (AT2G02010, AT5G17330, AT2G02000, AT1G65960, AT3G17760, and AT3G17720), one *GABA-T* gene (AT3G22200), and one *SSADH* gene (AT1G79440). Using the BLASTP 2.14 program, candidate genes for the *J. regia* GABA branch were initially identified. Hidden Markov models (PF00282, PF00202, and PF00171), corresponding to the GABA metabolic pathway gene families (GAD, GABA-T, and SSADH), were retrieved from the Pfam database [36] (https://pfam.xfam.org/, accessed on 7 May 2025), and HMM searches were conducted using the HMMER 3 software package. Based on the results of the BLASTP and HMM searches, redundant sequences were removed, and the final list of confirmed *J. regia* GABA metabolic pathway gene family members was compiled.

Protein sequence data for the confirmed gene family members were then uploaded to the Protein Parameter Calc module in TBtools 2.1 software to analyze their isoelectric points (pI), hydrophilicity characteristics, and molecular weight parameters [37]. Protein localization was predicted using WoLF PSORT (https://wolfpsort.hgc.jp/, accessed on 7 May 2025) [38], and protein conformations for the JrGABA gene family were determined using the SOPMA online resource (https://npsa.lyon.inserm.fr/cgi-bin/npsa_automat.pl?page=/NPSA/npsa_sopma.html, accessed on 7 May 2025).

### 2.3. Phylogenetic Study of the J. regia Genes Encoding GABA

The protein sequences of GABA pathway gene families, including GAD, GABA-T, and SSADH were retrieved from the NCBI database (https://www.ncbi.nlm.nih.gov/, accessed on 7 May 2025) from four plant species: *M. domestica*, *P. trichocarpa*, *A. thaliana*, and *J. regia*. Sequence alignment was performed using MEGA12, with the Maximum Likelihood method employed to construct the phylogenetic tree [39,40]. The resulting tree was visualized and enhanced using the iTOL online tool (https://itol.embl.de/, accessed on 8 May 2025) for better clarity and presentation [41].

### 2.4. Structure Analysis of J. regia GABA Branch Gene Family

The GABA gene family’s preserved patterns were identified via the MEME suite (https://meme-suite.org/meme/, accessed on 10 May 2025), limiting motif selection to up to ten. GABA pathway gene structure was assessed and displayed via the Amazing Optional Gene Viewer in TBtools [42].

### 2.5. Genomic Mapping and Synteny Assessment

The Amazing Gene Location From GTF/GFF module of TBtools 2.1 software was used to map the chromosome locations of GABA pathway-related gene family members [42]. To compare evolutionary relationships between dicot and monocot plants, cross-species collinearity analysis was performed with the MCScanX tool, utilizing the whole-genome protein sequences of *J. regia*, *A. thaliana* (representative dicots), and *Oryza sativa* (representative monocots) [43]. The Advanced Circos plug-in of TBtools was then employed to create a collinearity relationship map between these species, and tandem and segmental duplication events within chromosomes were analyzed. All visualization results were generated using TBtools software [37,42].

### 2.6. Cis-Regulatory Element Survey in Promoters

To identify the cis-acting elements within the 2000 bp promoter regions upstream of the genes of interest, were analyzed in the PlantCARE database (http://bioinformatics.psb.ugent.be/webtools/plantcare/html, accessed on 15 May 2025), leveraging the available genome annotation data. TBtools was then used to create a graphical representation of the analytical results [42].

### 2.7. Gene Expression Analysis

The same *J. regia* seedlings, as described in Section 2.1, were used to investigate salt and drought stress responses. Salt stress was induced by applying 200 mM NaCl solution through root irrigation, while drought stress was simulated using a 10% (*w*/*v*) PEG6000 solution, also via root irrigation [44]. Leaf samples were collected at three time points: before treatment (0 h), and 12 and 24 h post-treatment. The samples were immediately cryopreserved in liquid nitrogen and stored at −80 °C until analysis. For each time point, a single biological sample consisted of three seedlings, with three independent biological samples in total. All samples underwent transcriptome sequencing (RNA-seq) through Shanghai Haoweitai Biotechnology Co., Ltd. (Shanghai, China). Differential gene expression was determined using |log_2_FC| ≥ 1 alongside adjusted *p*-values below 0.05, following sequence analysis. The JrGABA gene’s relative abundance was quantified via FPKM, with logarithmic transformation (log_2_) applied for analysis and visualization via TBtools [42].

## 3. Results

### 3.1. Physicochemical Characterization of the GABA Pathway Gene Family in J. regia

A total of 39 GABA metabolic pathway-related gene family members were identified in *J. regia*, including 5 *GADs* genes (*JrGAD1-5*), 9 *GABA-Ts* genes (*JrGABA-T1-9*) and 25 *SSADHs* genes (*JrSSADH1-25*) (Table 1). Analysis of physicochemical properties revealed notable variability among the three gene families. JrGAD proteins ranged from 490 to 527 amino acids, with molecular weights of 55.13–57.97 kDa and theoretical isoelectric points (pI) from 5.59 to 8.29. JrGABA-T proteins exhibited a wider range, from 101 to 860 amino acids, with molecular masses of 11.06–94.73 kDa and pI values between 6.00 and 8.00. JrSSADH proteins showed the greatest diversity, with lengths from 130 to 1061 amino acids, molecular weights of 13.81–116.05 kDa, and pI values spanning 5.44–8.93. Predicted subcellular localization indicated that JrGAD2 and JrGAD3 are primarily associated with the cytoskeleton, while JrGAD4 and JrGAD5 localize to the cytoplasm, and JrGAD1 is found on the cell membrane. Over 30% of JrGABA-T members were predicted to localize in the peroxisome and chloroplast, with the remainder distributed across the mitochondria and cytoplasm. Approximately 40% of JrSSADH proteins were cytoplasmic, while around 20% each were localized to the chloroplast and mitochondria. The remaining proteins were predicted to reside in the nucleus, vacuole, Golgi apparatus, plasma membrane, peroxisome, or cytoskeleton.

### 3.2. Protein Secondary Structure Prediction for J. regia GABA Receptor Gene Family

Examination of the secondary structures of the GABA pathway gene family proteins GAD, GABA-T, and SSADH revealed that all members share four common structural elements: α-helices, β-turns, extended strands, and random coils (Table 2). In the GAD family proteins, α-helices accounted for 38.36–41.43%, random coils for 34.54–40.51%, extended strands for 12.90–15.92%, and β-turns for the smallest proportion, 4.93–6.73%. A comparative analysis of all three gene families showed a consistent pattern in the relative abundance of these structural components, following the order: α-helix > random coil > extended strand > β-turn.

### 3.3. Genetic Mapping of J. regia GABA-Related Genes

**Figure 1:**
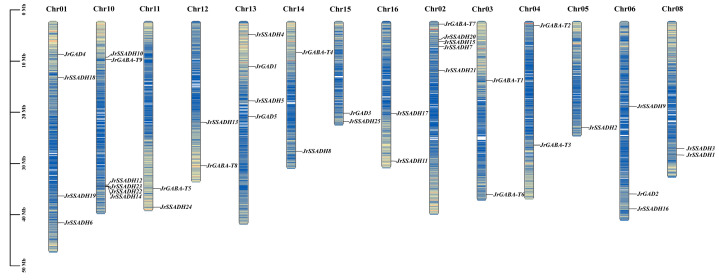
Chromosomal localization analysis of the *JrGABA* gene family.

Mapping and assessing the genomic positions of the three GABA metabolic pathway gene subfamilies clarified their chromosomal organization within the *J. regia* genome (Figure 1). In the *JrGADs* family, five genes were distributed across four chromosomes. Chromosome 13 contains two genes (*JrGAD1* and *JrGAD5*), while one gene was located on each of the remaining three chromosomes. The nine *JrGABA-T* genes were unevenly distributed across seven chromosomes, with two genes each located on chromosomes 3 and 4, and single genes present on chromosomes 2, 10, 11, 12, and 14. The *JrSSADHs* family comprising 25 members was distributed across12 chromosomes. Single genes were found on chromosomes 5, 11, 12, 14, and 15, whereas chromosome 10 harbored the largest number of members (five: *JrSSADH10*, *JrSSADH12*, *JrSSADH14*, *JrSSADH22*, and *JrSSADH23*). Additionally, four genes were mapped to chromosome 2, while chromosomes 6, 8, 13, and 16 each contained two *JrSSADH* genes.

### 3.4. Interspecific and Intraspecific Collinearity Analysis of J. regia GABA Branch Gene Family

**Figure 2:**
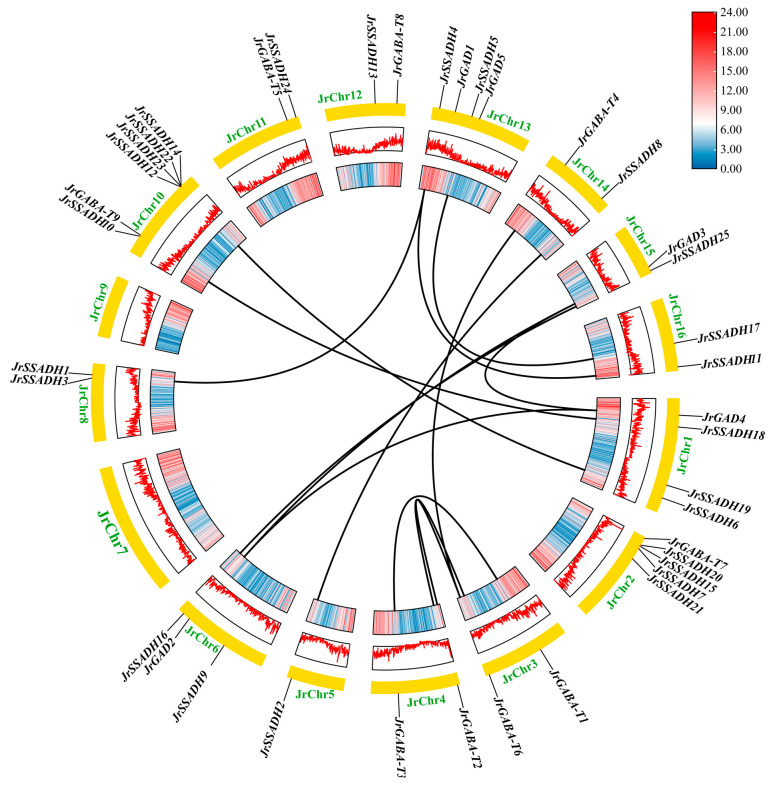
Intraspecific collinearity analysis of *JrGABA* genes in *J. regia*. The black lines within the circles represent the segmentally duplicated *JrGABA* gene pairs. The colored bar regions (red and blue) inside the chromosomes represent different genomic features (gene density, GC content). The yellow regions marked at the edges represent the chromosomes.

Collinearity analysis of the *J. regia* GABA pathway gene family revealed 15 duplication events across the GAD, GABA-T, and SSADH subfamilies (Figure 2). These repetitive gene pairs were located on 12 different chromosomes (JrChr 1, 3, 4, 5, 6, 8, 10, 13, 14, 15, and 16), demonstrating widespread genomic distribution of these conserved sequences. No tandem repeat events were detected on JrChr 2, JrChr 7, JrChr 9, JrChr 11 and JrChr 12. Notably, at least two duplication events occurred between JrChr 3 and JrChr 4, JrChr 1 and JrChr 10, and JrChr 13 and JrChr 16. These duplication patterns likely contributed to the expansion and functional diversification of the *JrGABA* gene family in *J. regia*.

**Figure 3:**
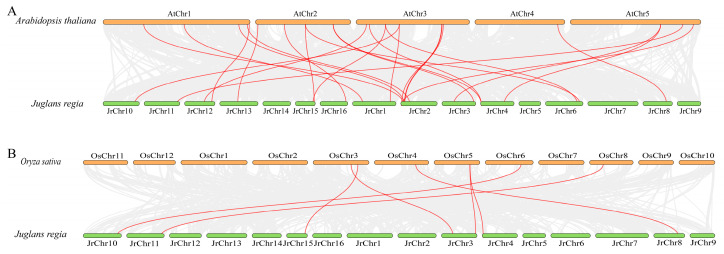
Interspecific collinearity analysis of GABA shunt genes in genomes of different species. (**A**) The collinearity between *J. regia* and *A. thaliana*. (**B**) The collinearity between *J. regia* and *O. sativa*.

Comparative collinearity analysis between *J. regia* and *A. thaliana* as well as *O. sativa* further elucidated the evolutionary conservation of the GABA metabolic pathway. A total of 26 collinear segments were identified between *J. regia* (12 chromosomes) and *A. thaliana* (5 chromosomes) (Figure 3A), with JrChr 2 exhibiting the highest number of homologous gene pairs (seven). In contrast, *J. regia* (6 chromosomes) and *O. sativa* (5 chromosomes) shared only seven collinear fragment pairs (Figure 3B), with JrChr 3 showing the highest degree of correspondence (two pairs). Overall, the number of *J. regia* to *A. thaliana* orthologous gene pairs (26) was considerably higher than that of *J. regia*–*O. sativa* (7), suggesting that *J. regia* shares a closer evolutionary relationship with *A. thaliana*.

### 3.5. Evolutionary Analysis of J. regia GABA Branch Gene Family

**Figure 4:**
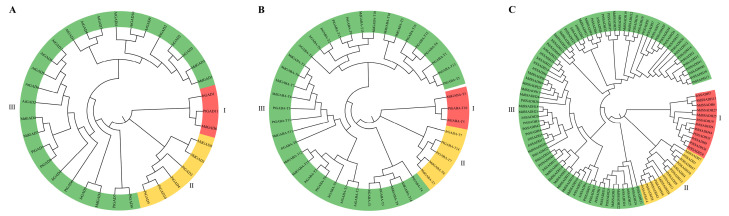
Phylogenetic tree of the *GABA* gene family: (**A**) the phylogenetic tree of the *GAD* gene family, (**B**) the phylogenetic tree of the *GABA-T* gene family, and (**C**) the phylogenetic tree of the *SSADH* gene family. Among them, At—*A. thaliana*; Jr—*J. regia*; Md—*M. domestica*; and Pt—*P. trichocarpa*.

To elucidate the evolutionary relationships of the GABA metabolic pathway gene family in *J. regia*, protein sequences from four species *M. domestica*, *P. trichocarpa*, *A. thaliana*, and *J. regia* were used to construct phylogenetic trees. This analysis provided insights into the evolutionary connections and divergence patterns among gene family members. The GABA-related genes were grouped into three distinct clusters: Cluster I, Cluster II, and Cluster III. In the *GAD* subfamily (Figure 4A), *MdGAD5* and *JrGAD5* formed a closely related branch, indicating strong sequence similarity and a close evolutionary relationship, suggesting functional conservation. In contrast, *MdGAD6*, *PtGAD11*, and *JrGAD1* were distributed on separate branches, reflecting an early evolutionary divergence. Within the *GABA-T* subfamily (Figure 4B), *PtGABA-T11*, *MdGABA-T11*, and *JrGABA-T8* clustered tightly together, indicating a close genetic relationship and potential conservation of function. In the *SSADH* subfamily (Figure 4C), *MdSSADH13*, *MdSSADH6*, and *JrSSADH7* in subgroup I were located on a closely connected terminal branch, suggesting high sequence similarity and a likely shared evolutionary origin within a rapidly evolving subgroup.

### 3.6. Analysis of Conserved Motifs and Gene Architecture in J. regia GABA Genes

**Figure 5:**
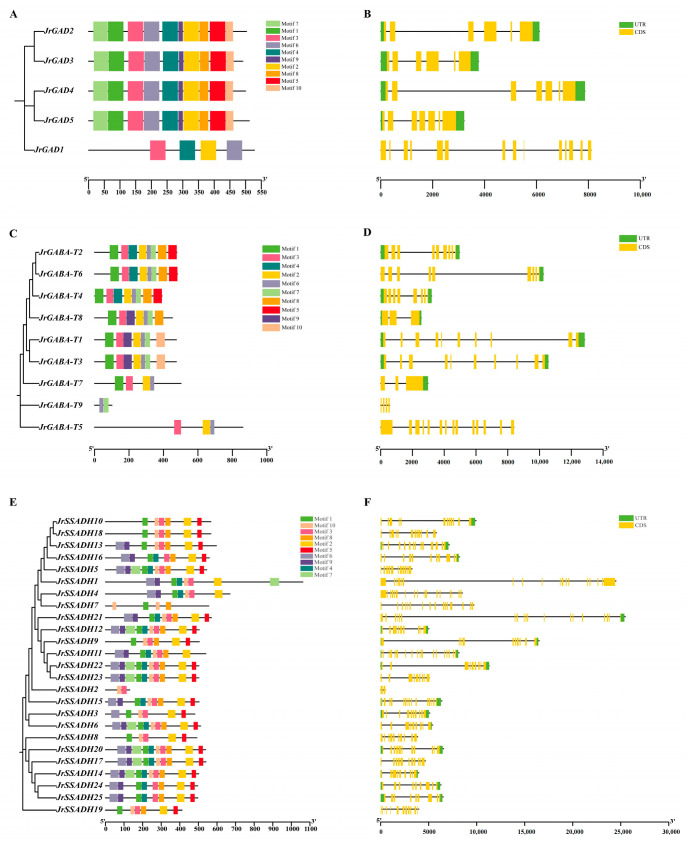
Analysis of conserved motifs and gene structures of *JrGABAs*. (**A**) Motif composition of the *JrGAD* gene family. Ten motifs are shown as boxes in different colors. (**B**) Gene structures of *JrGADs*. (**C**) Motif composition of the *JrGABA-Ts* gene family. Ten motifs are displayed as boxes in different colors. (**D**) Gene structures of the *JrGABA-Ts* gene family. (**E**) Motif composition of the *JrSSADHs* gene family. Ten motifs are shown as boxes in different colors. (**F**) Gene structures of the *JrSSADHs* gene family. Green and yellow boxes represent UTR and CDS regions, respectively, and black lines indicate introns.

To understand the genomic structure of *GABA* metabolism genes, a comprehensive analysis was performed on its conserved motifs and exon-intron organizational patterns. The conserved motif analysis revealed that *JrGAD2, JrGAD3*, *JrGAD4*, and *JrGAD5* shared identical motifs, with all 10 identified motifs (Motif 1–10) indicating highly conserved sequences (Figure 5A). In contrast, *JrGAD1* exhibited a simpler motif composition, containing only Motifs 3, 4, 2, and 6. Exon-intron structure analysis showed that the coding regions (CDS) of *JrGAD2* and *JrGAD3* were similar in both number and organization, with short, conservative untranslated regions (UTRs). However, *JrGAD1′*s CDS structure was more complex, with numerous exons distributed across the gene and UTRs either missing or significantly shortened in some regions (Figure 5B).

For the *JrGABA-T* gene family, conserved motifs were highly consistent across *JrGABA-T2*, *JrGABA-T6*, and *JrGABA-T4*, with 8 motifs (Motif 1–8) identified, indicating sequence conservation (Figure 5C). *JrGABA-T5* contained Motifs 3, 2, and 6, while *JrGABA-T9* included Motifs 6 and 7. The CDS of *JrGABA-T6*, *JrGABA-T1*, and *JrGABA-T3* were long and dispersed, while *JrGABA-T9* had fewer, more concentrated CDS, suggesting evolutionary diversity and functional divergence within this gene family (Figure 5D).

In the *JrSSADH* gene family, most members, such as *JrSSADH12*, *JrSSADH22*, *JrSSADH23*, and *JrSSADH6*, exhibited highly consistent motifs (Motif 1–10), while *JrSSADH2* was distinct, containing only Motifs 3 and 10 (Figure 5E). Exon-intron analysis revealed that the CDS of genes like *JrSSADH1*, *JrSSADH21*, *JrSSADH9*, and *JrSSADH22* were long and scattered, with complex UTR distributions. *JrSSADH2*, on the other hand, had a shorter CDS and a smaller proportion of UTR (Figure 5F). These results highlight significant variations in the genomic architecture of the *JrGABA-T* and *JrSSADH* gene families, suggesting both evolutionary diversity and functional divergence.

### 3.7. Promoter Cis-Acting Element Analysis of GABA Branch Gene Family in J. regia

**Figure 6:**
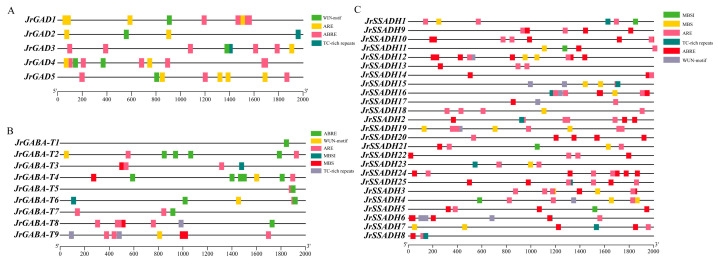
(**A**) Prediction and analysis of cis-acting elements in members of the *JrGADs* gene family. (**B**) Prediction and analysis of cis-acting elements in members of the *JrGABA-Ts* gene family. (**C**) Prediction and analysis of cis-acting elements in members of the *JrSSADHs* gene family.

To define control mechanisms in the *J. regia GABA* gene family, cis-element analysis was performed on promoter sequences of its constituent genes. The results revealed that cis-acting elements associated with hormonal and stress responses are prevalent in the promoter regions of all *GABA* gene family members. In the *GAD* family (Figure 6A), TC-rich repeats important for stress response and defense were notably rare, appearing only in the promoters of *JrGAD2* and *JrGAD5*. The most common elements were ABRE (abscisic acid response element) and ARE (anaerobic response element), which together accounted for 25–34% of the total elements. In the *GABA-T* family (Figure 6B), ABRE elements were the most abundant, comprising 36% of the total, followed by ARE elements at 32%, with WUN-motif and TC-rich repeats each representing 8%. In the *SSADH* family (Figure 6C), ABRE elements were also the most frequent, making up over 35%, followed closely by ARE elements at 34%, while MBSI (MYB binding site I) elements were the least common at just 3%. These findings indicate that the promoter regions of the *J. regia GABA* gene family are rich in stress and hormone-responsive elements, reflecting the distinct roles of different gene family members in stress response and evolution.

### 3.8. Expression Profiling of J. regia GABA Pathway Genes During Abiotic Stress

**Figure 7:**
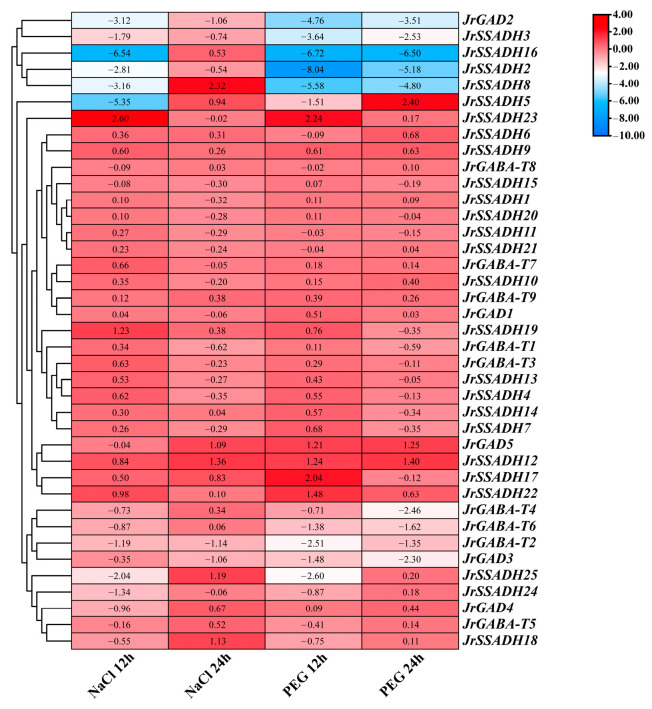
Expression patterns of gene family members in GABA shunt pathway of *J*. *regia* under abiotic stress.

To define *JrGABA* pathway gene behavior in *J. regia* trees facing salinity and dehydration, transcriptomic profiling was conducted. The data revealed (Figure 7) revealed a significant increase in the expression of *JrSSADH23*, *JrSSADH17*, and *JrSSADH12* within 12 h of stress exposure, with expression levels increased by 2.24–2.60, 0.50–2.04, and 0.98–1.48-fold, respectively, compared to the control group. At 24 h post-treatment, the expression of *JrSSADH5*, *JrGAD5*, and *JrSSADH12* increased by 0.94–2.24, 1.09–1.25, and 1.36–1.40-fold, respectively. The transcript levels of *JrSSADH5*, *JrGAD5*, and *JrSSADH12* increased by 0.94–2.24, 1.09–1.25, and 1.36–1.40 fold, respectively, at 24 h post-treatment compared to controls. Conversely, after a 12 h treatment, the expression levels of *JrGAD2*, *JrSSADH16*, *JrSSADH2*, and *JrSSADH8* decreased by half seen in the control group. Even after 24 h, *JrGAD2*, *JrSSADH3*, and *JrSSADH2* expression remained decreased across both treatments by 1.06 to 3.51, 0.74 to 2.53, and 0.54 to 5.18 times the control group, respectively. Additionally, *JrSSADH9*, *JrSSADH12*, *JrSSADH22*, and *JrGABA-T9* showed increased expression across all measured intervals under both salt and drought stress.

## 4. Discussion

GABA transcription factors are found throughout a broad range of plant species, playing a crucial role in essential biological functions related to growth and development. These factors influence vital processes, including hormonal signaling pathways and regulation of carbon and nitrogen metabolism [12]. The GABA metabolic pathway is primarily regulated by three key enzymes, GAD, GABA-T, and SSADH, which serve as the linchpins of this regulation [12]. The GABA metabolism gene family has been extensively characterized various plant species, including the GAD gene family in broccoli buds [45], *A. thaliana* [46] and *P. ginseng* [19], the GABA-T gene family of *O. sativa* [47], *Brassica napus* [48] and *Morus alba* [49], and the SSADH gene family of *Musa acuminata* [50].

In this current study, a total of 5 GAD genes, 9 GABA-T genes, and 25 SSADH genes were identified through genome-wide analysis of *J. regia*. Analysis of their physicochemical properties revealed that the majority of proteins in the GABA metabolic pathway family exhibit hydrophilic characteristics, with members of the GAD protein family showing no hydrophobic properties. In *P. trichocarpa*, the PopGAD gene family shows similar trends with the *J. regia JrGAD* gene family in terms of protein hydrophobicity and stability. This suggests that, despite variations in sequence and number across different plant species, the biological roles of the GAD gene family remain consistent throughout various stages of plant growth and development [34]. Subcellular localization analysis revealed that 94% of the genes in *M. acuminata* are predominantly localized in mitochondria and the cytoplasm, indicating that their functions are closely tied to these core cellular regions [50]. The *SSADH* genes in *J. regia* are widely distributed, suggesting potential functional diversity, including roles in environmental stress responses, signal transduction, and regulation of growth and development [51].

The exon-intron structure is significant for the evolution of gene families, influencing gene function and species adaptability [52]. Within the GABA metabolic pathway gene family of *J. regia*, the conservation of motif and gene structure of the same subfamily members is not an isolated phenomenon. For instance, he alignment of amino acid sequences within the *G. hirsutum* ORP gene family demonstrates that genes within shared subgroups exhibit strong similarities in exon-intron structure and conserved motif composition [53]. Motif similarity in the GABA branch gene family may be involved in the same metabolic pathway, as conserved motifs are often associated with similar structures and functional domains involved in biochemical reactions or molecular interactions. In the absence of calmodulin (CaM), the crystal structure of *A. thaliana GAD1* retains the decarboxylation process that converts glutamate to GABA, relying on conserved motifs and structural features. GABA, in turn, regulates cell osmotic pressure, enhances water retention, and helps plants resist environmental stress [54]. The JrGAD (subgroup I) of *J. regia* contains a single JrGAD gene, forming a unique functional branch, similar to *B. oleracea BoGAD5*, which is associated with GABA enrichment [45]. This functional divergence may represent the adaptability of plants to adapt to specific physiological environment needs during evolution. GABA-T genes, located in subgroups I–III, exhibit functional divergence resulting from prolonged evolution, enabling adaptation to intricate environmental shifts. Studies have shown that members of the same branch of the evolutionary tree of the GABA-T gene family in *B. napus* are co-expressed under salt stress, and its conserved PLP binding motif ensures GABA degradation efficiency, thereby maintaining cell osmotic pressure stability [48,55]. Among the members of *JrSSADH* gene in *J. regia*, most of the conserved motif members of the gene have the same motif, which is similar to the SSADH gene family of *M. domestica* and *G. hirsutum*. Lineage members in the phylogeny exhibit correlated expression patterns during stress. The conserved structural motifs guarantee catalytic activity, efficiently promote GABA branch metabolism, maintain cell osmotic pressure, and help plants adapt to low temperature and drought stress environments [32,51].

Collinearity analysis between *J. regia*, *A. thaliana*, and *O. sativa* revealed that 26 pairs of genes in the *J. regia* GABA family formed interspecies collinearity pairs with *A. thaliana*, primarily through tandem and fragment repetitions, while only 7 pairs showed homology with *O. sativa*. This suggests a higher degree of evolutionary conservation between the *J. regia* GABA gene family and the *Arabidopsis* GABA family, which may be crucial for plant functions in stress responses and development. The widespread distribution of *JrGABA* genes across *J. regia* chromosomes may help the plant adapt to and survive environmental stressors such as drought, salinity, and pathogen invasion by modulating gene expression. Cis-acting elements, often referred to as “molecular switches” in gene regulation, are key in mediating the response of genes in the GABA metabolic pathway to environmental stress [56]. In *A. thaliana*, drought stress increases ABA content in cells, which binds to receptors and activates downstream signaling pathways. AREB and ABF transcription factors are phosphorylated and bind to ABRE elements, inducing drought-responsive gene expression, promoting the synthesis of osmotic regulators like proline, and enhancing drought tolerance [57]. All genes in the GABA metabolic pathway contain defense- and stress-related components, further highlighting their role in enhancing plant resistance to adverse environmental conditions.

The JrGABA pathway genes in *J. regia* demonstrate varied responses to salt and drought stress, playing key roles in maintaining osmotic balance and supporting energy metabolism in plants. Under NaCl treatment, *JrGAD5* showed sustained high expression, similar to the expression pattern of *GhGAD6* under Cd^2+^ stress in *G. hirsutum* [58]. In *Solanum lycopersicum*, the amplification of the *SlGAD1* gene increased GABA levels and reduced accumulation of ROS under salt stress. Silencing SlGAD1 further confirmed its positive effect on GABA biosynthesis and improved salt tolerance in tomato [59], emphasizing the importance of GAD expression in maintaining stress resistance. Additionally, *JrSSADH23* expression increased under both salt and drought stress, suggesting it may be an early responder, adjusting succinate-semialdehyde metabolism to provide an energy boost when stress hits. Overexpressing *MaSSADH1-14* genes from *M. acuminata* in tobacco plants enhanced GABA accumulation and salt tolerance [50]. Overall, the JrGABA pathway genes in *J. regia* exhibit diverse expression control mechanisms that contribute to stress adaptation and response.

## 5. Conclusions

In conclusion, this research provides a comprehensive analysis of the structural characteristics and evolutionary relationships among three gene families—GAD, GABA-T, and SSADH—that are involved in the GABA metabolism of *J. regia*. A total of 39 distinct members across these families were identified. The regulatory regions of all genes discovered contained stress-related sequences, particularly those associated with desiccation and high salinity, highlighting the critical role of the GABA metabolic pathway in regulating plant growth, development, and stress responses. Evolutionary analysis suggests that the expansion of these gene families is primarily driven by gene duplication events, including segmental duplication. Some members of the JrGABA gene family displayed distinct and significant expression patterns under abiotic stress and hormone treatments, with *JrSSADH23* showing strong expression profiles in both saline and drought conditions. This study lays the groundwork for future research on the GABA pathway’s role in the response of *J. regia* to abiotic stressors.

## Figures and Tables

**Table 1:** The length distribution and physicochemical properties of *J. regia* GABA proteins.

Gene ID	Gene Name	Number of Amino Acid	Molecular Weight	Theoretical pI	Instability Index	Aliphatic Index	Grand Average of Hydropathicity	Predicted Location(s)
LOC109003131	*JrGAD1*	527	57,972.18	8.29	41.26	95.43	−0.02	plas
LOC109012088	*JrGAD2*	502	56,537.07	5.91	37.29	89.08	−0.172	cysk
LOC108993168	*JrGAD3*	490	55,133.44	5.78	40.77	89.33	−0.167	cysk
LOC108988130	*JrGAD4*	499	56,626.09	5.62	31.54	90.02	−0.23	cyto
LOC108997089	*JrGAD5*	511	57,674.08	5.59	35.41	89.45	−0.255	cyto
LOC109011059	*JrGABA-T1*	474	52,156.99	6.65	36.02	89.32	−0.172	mito
LOC108990021	*JrGABA-T2*	477	51,739.45	6.43	32.11	89.12	0.004	pero
LOC108990262	*JrGABA-T3*	474	52,020.95	6.8	35.87	91.81	−0.155	chlo
LOC108996966	*JrGABA-T4*	391	42,294.75	6.31	21.71	92.79	0.099	pero
LOC108998892	*JrGABA-T5*	860	94,738.09	6.08	41.52	85.99	−0.061	chlo
LOC108985282	*JrGABA-T6*	481	52,078.73	7.59	34.87	86.15	−0.062	pero
LOC108998090	*JrGABA-T7*	501	53,932.96	7.23	39.28	81.06	−0.086	chlo
LOC109009895	*JrGABA-T8*	452	48,609.57	6.43	38.76	94.76	0.032	chlo
LOC108996782	*JrGABA-T9*	101	11,061.99	6.8	41.12	108.02	0.489	cyto
LOC108986827	*JrSSADH1*	1061	116,054.54	7.29	51.31	75.57	−0.391	nucl
LOC108985392	*JrSSADH2*	130	13,817.1	8.84	33.99	104.31	0.272	chlo
LOC108983652	*JrSSADH3*	480	53,271.88	8.38	40.77	101.6	−0.009	cysk
LOC108983908	*JrSSADH4*	669	73,056.89	8.83	38.28	87.13	−0.182	cyto
LOC118344148	*JrSSADH5*	544	59,635.3	6.3	29.66	87.72	−0.135	chlo
LOC108992687	*JrSSADH6*	511	55,778.21	5.81	29.94	90.84	−0.048	cyto
LOC108999249	*JrSSADH7*	555	61,844.14	6.48	37.56	89.24	−0.151	mito
LOC108992688	*JrSSADH8*	503	54,693.07	5.57	30.5	92.15	0.013	cysk
LOC108998675	*JrSSADH9*	491	54,689.42	8.93	43.44	95.78	−0.055	cyto
LOC108994687	*JrSSADH10*	504	54,974.99	7.66	35.06	104.64	0.122	plas
LOC108983383	*JrSSADH11*	566	62,522.22	8.11	37.37	98.89	0.002	chlo
LOC108995743	*JrSSADH12*	539	57,994.52	8.27	39.16	85.96	−0.044	mito
LOC109004656	*JrSSADH13*	504	54,805.97	6.11	28.73	93.89	−0.021	cyto
LOC108994823	*JrSSADH14*	596	66,033.3	7.18	38.99	91.12	0.015	vacu
LOC109004652	*JrSSADH15*	501	54,194.62	5.99	25.18	94.05	0.064	cyto
LOC108999250	*JrSSADH16*	503	54,539.68	5.44	27.77	93.34	−0.01	pero
LOC109012892	*JrSSADH17*	558	60,823.29	8.05	45.82	88.75	−0.109	cyto
LOC108996365	*JrSSADH18*	566	62,483.16	8.53	38.45	94.75	−0.091	chlo
LOC109009030	*JrSSADH19*	411	44,362.14	6.04	33.54	92.26	0.106	chlo
LOC108999220	*JrSSADH20*	538	58,649.98	7.59	33.54	89.11	−0.074	mito
LOC108983566	*JrSSADH21*	569	61,110.39	8.63	33.67	93.25	−0.032	mito
LOC109004654	*JrSSADH22*	502	54,439.69	5.83	26.94	92.53	0.029	cyto
LOC108997881	*JrSSADH23*	502	54,422.71	6	27.59	93.86	0.027	cyto
LOC109005232	*JrSSADH24*	496	53,190.69	7.08	37.99	94.33	0.053	cyto
LOC109000833	*JrSSADH25*	496	53,276.73	7.47	39.32	91.41	0.015	cyto

**Table 2:** Prediction of protein secondary structure of the GABA gene family.

Gene Name	Alpha Helix (Hh)	Extended Strand (Ee)	Beta Turn (Tt)	Random Coil (Cc)
*JrGAD1*	47.63%	12.90%	4.93%	34.54%
*JrGAD2*	41.43%	13.55%	5.58%	39.44%
*JrGAD3*	40.41%	15.92%	6.73%	36.94%
*JrGAD4*	40.48%	14.23%	6.01%	39.28%
*JrGAD5*	38.36%	15.07%	6.07%	40.51%
*JrGABA-T1*	38.19%	17.09%	10.76%	33.97%
*JrGABA-T2*	40.25%	17.61%	7.76%	34.38%
*JrGABA-T3*	38.19%	15.82%	9.92%	36.08%
*JrGABA-T4*	41.18%	17.90%	8.95%	31.97%
*JrGABA-T5*	39.88%	13.14%	5.23%	41.74%
*JrGABA-T6*	42.62%	16.22%	8.32%	32.85%
*JrGABA-T7*	40.52%	18.36%	9.18%	31.94%
*JrGABA-T8*	40.04%	19.47%	9.07%	31.42%
*JrGABA-T9*	41.58%	15.84%	13.86%	28.71%
*JrSSADH1*	24.98%	15.55%	7.26%	52.21%
*JrSSADH2*	46.92%	18.46%	10.00%	24.62%
*JrSSADH3*	44.79%	15.62%	6.67%	32.92%
*JrSSADH4*	36.17%	17.34%	7.47%	39.01%
*JrSSADH5*	40.81%	15.81%	7.90%	35.48%
*JrSSADH6*	40.12%	18.20%	8.02%	33.66%
*JrSSADH7*	38.74%	15.86%	5.23%	40.18%
*JrSSADH8*	44.40%	16.90%	7.74%	30.96%
*JrSSADH9*	47.02%	15.87%	6.35%	30.75%
*JrSSADH10*	40.46%	18.90%	6.36%	34.28%
*JrSSADH11*	38.59%	18.00%	7.98%	35.44%
*JrSSADH12*	40.08%	18.06%	9.13%	32.74%
*JrSSADH13*	42.79%	12.75%	6.54%	37.92%
*JrSSADH14*	39.52%	18.76%	7.78%	33.93%
*JrSSADH15*	43.74%	15.90%	6.76%	33.60%
*JrSSADH16*	42.65%	16.67%	6.63%	34.05%
*JrSSADH17*	41.48%	16.11%	7.78%	34.63%
*JrSSADH18*	42.93%	15.72%	7.07%	34.28%
*JrSSADH19*	38.20%	20.19%	6.08%	35.52%
*JrSSADH20*	41.08%	16.91%	8.18%	33.83%
*JrSSADH21*	48.15%	14.06%	7.73%	30.05%
*JrSSADH22*	41.04%	17.53%	6.97%	34.46%
*JrSSADH23*	40.95%	17.69%	6.76%	34.50%
*JrSSADH24*	40.73%	18.55%	6.05%	34.68%
*JrSSADH25*	41.33%	18.55%	6.25%	33.87%

## Data Availability

The data that support the findings of this study are available from the corresponding author upon reasonable request.

## References

[1] Zhang H., Zhu J., Gong Z., Zhu J.K. (2022). Abiotic stress responses in plants. Nat. Rev. Genet..

[2] Hussain M., Farooq S., Hasan W., Ul-Allah S., Tanveer M., Farooq M., Nawaz A. (2018). Drought stress in sunflower: Physiological effects and its management through breeding and agronomic alternatives. Agric. Water Manag..

[3] Fu Z.W., Feng Y.R., Gao X., Ding F., Li J.H., Yuan T.T., Lu Y.T. (2023). Salt stress-induced chloroplastic hydrogen peroxide stimulates pdTPI sulfenylation and methylglyoxal accumulation. Plant Cell.

[4] Jan R., Asaf S., Numan M., Lubna, Kim K.-M. (2021). Plant secondary metabolite biosynthesis and transcriptional regulation in response to biotic and abiotic stress conditions. Agronomy.

[5] Gabira M.M., Bergeron Y., Duarte M.M., Aguiar N.S.d., Kratz D., Silva M.R.d., Wendling I., Girona M.M. (2024). Morphological, physiological, and biochemical responses of yerba mate (*Ilex paraguariensis*) genotypes to water deficit. New For..

[6] Wang K., Yang M., Wu S., Liu Q., Cao S., Chen W., Shi L. (2022). Identification of laccase gene family members in peach and its relationship with chilling induced browning. Chin. J. Biotechnol..

[7] Hu Y., Huang X., Xiao Q., Wu X., Tian Q., Ma W., Shoaib N., Liu Y., Zhao H., Feng Z. (2024). Advances in plant GABA research: Biological functions, synthesis mechanisms and regulatory pathways. Plants.

[8] Zhang Y.Y. (2016). γ-Aminobutyric acid (GABA) in Fresh-Cut fruits and Vegetables. Master’s Thesis.

[9] Ramesh S.A., Tyerman S.D., Gilliham M., Xu B. (2017). γ-aminobutyric acid (GABA) signalling in plants. Cell. Mol. Life Sci..

[10] Ramos-Ruiz R., Martinez F., Knauf-Beiter G. (2019). The effects of GABA in plants. Cogent Food Agric..

[11] Hayat F., Khan U., Li J., Ahmed N., Khanum F., Iqbal S., Altaf M.A., Ahmad J., Javed H.U., Peng Y. (2023). γ aminobutyric acid (GABA): A key player in alleviating abiotic stress resistance in horticultural crops: Current insights and future directions. Horticulturae.

[12] Ahmad S., Fariduddin Q. (2024). Deciphering the enigmatic role of gamma-aminobutyric acid (GABA) in plants: Synthesis, transport, regulation, signaling, and biological roles in interaction with growth regulators and abiotic stresses. Plant Physiol. Biochem..

[13] Jiang X., Xu Q., Zhang A., Liu Y., Zhao L., Gu L., Yuan J., Jia H., Shen X., Li Z. (2021). Optimization of γ-aminobutyric acid (GABA) accumulation in germinating adzuki beans (*Vigna angularis*) by vacuum treatment and monosodium glutamate, and the molecular mechanisms. Front. Nutr..

[14] Bown A.W., Shelp B.J. (2020). Does the GABA shunt regulate cytosolic GABA?. Trends Plant Sci..

[15] Yin Y., Cheng C., Fang W. (2018). Effects of the inhibitor of glutamate decarboxylase on the development and GABA accumulation in germinating fava beans under hypoxia-NaCl stress. RSC Adv..

[16] Zhou M., Hassan M.J., Peng Y., Liu L., Liu W., Zhang Y., Li Z. (2021). γ-aminobutyric acid (GABA) priming improves seed germination and seedling stress tolerance associated with enhanced antioxidant metabolism, DREB expression, and dehydrin accumulation in white clover under water stress. Front. Plant Sci..

[17] AL-Quraan N.A., Sartawe F.A., Qaryouti M.M. (2013). Characterization of γ-aminobutyric acid metabolism and oxidative damage in wheat (*Triticum aestivum* L.) seedlings under salt and osmotic stress. J. Plant Physiol..

[18] Akçay N., Bor M., Karabudak T., Özdemir F., Türkan İ. (2012). Contribution of gamma amino butyric acid (GABA) to salt stress responses of *Nicotiana sylvestris* CMSII mutant and wild type plants. J. Plant Physiol..

[19] Lee J.H., Kim Y.J., Jeong D.Y., Sathiyaraj G., Pulla R.K., Shim J.S., In J.G., Yang D.C. (2010). Isolation and characterization of a *glutamate decarboxylase* (*GAD*) gene and their differential expression in response to abiotic stresses from *Panax ginseng* C. A. Meyer. Mol. Biol. Rep..

[20] Carillo P. (2018). GABA shunt in durum Wheat. Front. Plant Sci..

[21] Jalil S.U., Ahmad I., Ansari M.I. (2017). Functional loss of GABA transaminase (GABA-T) expressed early leaf senescence under various stress conditions in *Arabidopsis thaliana*. Curr. Plant Biol..

[22] Renault H., Roussel V., El Amrani A., Arzel M., Renault D., Bouchereau A., Deleu C. (2010). The *Arabidopsis pop2-1* mutant reveals the involvement of GABA transaminase in salt stress tolerance. BMC Plant Biol..

[23] Menduti G., Vitaliti A., Capo C.R., Lettieri-Barbato D., Aquilano K., Malaspina P., Rossi L. (2020). SSADH variants increase susceptibility of U87 cells to mitochondrial pro-oxidant insult. Int. J. Mol. Sci..

[24] Bouche N. (2003). GABA signaling: A conserved and ubiquitous mechanism. Trends Cell Biol..

[25] Ma Y., Wang P., Chen Z., Gu Z., Yang R. (2018). GABA enhances physio-biochemical metabolism and antioxidant capacity of germinated hulless barley under NaCl stress. J. Plant Physiol..

[26] AL-Quraan N.A., Samarah N.H., AL-Fawaz A.M. (2024). The physiological effect of GABA priming on pepper (*Capsicum annuum* L.) during seed germination under salt stress. Plant Growth Regul..

[27] Rezaei-Chiyaneh E., Seyyedi S.M., Ebrahimian E., Moghaddam S.S., Damalas C.A. (2018). Exogenous application of gamma-aminobutyric acid (GABA) alleviates the effect of water deficit stress in black cumin (*Nigella sativa* L.). Ind. Crops Prod..

[28] Guo W., Chen J., Li J., Huang J., Wang Z., Lim K.J. (2020). Portal of Juglandaceae: A comprehensive platform for Juglandaceae study. Hortic. Res..

[29] Keller I., Rodrigues C.M., Neuhaus H.E., Pommerrenig B. (2021). Improved resource allocation and stabilization of yield under abiotic stress. J. Plant Physiol..

[30] Jiang Z., Van Zanten M., Sasidharan R. (2025). Mechanisms of plant acclimation to multiple abiotic stresses. Commun. Biol..

[31] Abdullah W., Wani K.I., Naeem M., Aftab T. (2025). From neurotransmitter to plant protector: The intricate world of GABA signaling and its diverse functions in stress mitigation. J. Plant Growth Regul..

[32] Zheng J., Zhang Z., Zhang N., Liang Y., Gong Z., Wang J., Ditta A., Sang Z., Wang J., Li X. (2024). Identification and function analysis of GABA branch three gene families in the cotton related to abiotic stresses. BMC Plant Biol..

[33] Zheng Q., Su S., Wang Z., Wang Y., Xu X. (2021). Comprehensive genome-wide identification and transcript profiling of GABA pathway gene family in apple (*Malus domestica*). Genes.

[34] Chen W., Cheng T.L., Ji J., Wu Y.Y., Xie T.T., Jiang Z.P. (2020). Identification of three gene families in the GABA shunt and their expression analysis in poplar. J. Nanjing For. Univ./Nat. Sci. Ed..

[35] Dove A. (2000). *Arabidopsis* database. Nat. Biotechnol..

[36] Mistry J., Chuguransky S., Williams L., Qureshi M., Salazar G.A., Sonnhammer E.L.L., Tosatto S.C.E., Paladin L., Raj S., Richardson L.J. (2021). Pfam: The protein families database in 2021. Nucleic Acids Res..

[37] Chen C., Chen H., Zhang Y., Thomas H.R., Frank M.H., He Y., Xia R. (2020). TBtools: An integrative toolkit developed for interactive analyses of big biological data. Mol. Plant.

[38] Chou K.C., Shen H.B. (2010). Plant-mPLoc: A top-down strategy to augment the power for predicting plant protein subcellular localization. PLoS ONE.

[39] Tamura K., Stecher G., Kumar S. (2021). MEGA11: Molecular evolutionary genetics analysis version 11. Mol. Biol. Evol..

[40] Kumar S., Stecher G., Suleski M., Sanderford M., Sharma S., Tamura K. (2024). MEGA12: Molecular evolutionary genetic analysis version 12 for adaptive and green computing. Mol. Biol. Evol..

[41] Letunic I., Bork P. (2021). Interactive tree of life (iTOL) v5: An online tool for phylogenetic tree display and annotation. Nucleic Acids Res..

[42] Chen C., Wu Y., Li J., Wang X., Zeng Z., Xu J., Liu Y., Feng J., Chen H., He Y. (2023). TBtools-II: A “one for all, all for one” bioinformatics platform for biological big-data mining. Mol. Plant.

[43] Wang Y., Tang H., Wang X., Sun Y., Joseph P.V., Paterson A.H. (2023). Detection of colinear blocks and synteny and evolutionary analyses based on utilization of MCScanX. Nat. Protoc..

[44] Li J., Zhou H., Xiong C., Peng Z., Du W., Li H., Wang L., Ruan C. (2022). Genome-wide analysis R2R3-MYB transcription factors in *Xanthoceras sorbifolium* Bunge and functional analysis of XsMYB30 in drought and salt stresses tolerance. Ind. Crop. Prod..

[45] Jiao W.H., Wei Q.L., Chen J.H., Cao S.F., Luo M., Qian Y., Chen Y., Wei Y.Y., Shao X.F., Xu F. (2024). Identification analysis of *GAD* gene family, and the role of *BoGAD5* in GABA enrichment in broccoli sprouts. Plant Growth Regul..

[46] Benidickson K.H., Raytek L.M., Hoover G.J., Flaherty E.J., Shelp B.J., Snedden W.A., Plaxton W.C. (2023). Glutamate decarboxylase-1 is essential for efficient acclimation of *Arabidopsis thaliana* to nutritional phosphorus deprivation. New Phytol..

[47] Shimajiri Y., Ozaki K., Kainou K., Akama K. (2013). Differential subcellular localization, enzymatic properties and expression patterns of γ-aminobutyric acid transaminases (GABA-ts) in rice (*Oryza sativa*). J. Plant Physiol..

[48] Faës P., Niogret M.F., Montes E., Cahérec F.L., Bouchereau A., Deleu C. (2015). Transcriptional profiling of genes encoding GABA-transaminases in *Brassica napus* reveals their regulation by water deficit. Environ. Exp. Bot..

[49] Zhang M., Liu Z., Fan Y., Liu C., Wang H., Li Y., Xin Y., Gai Y., Ji X. (2022). Characterization of GABA-transaminase gene from mulberry (*Morus multicaulis*) and its role in salt stress tolerance. Genes.

[50] Guo X., Yang F., Zhang X., Tang M., Wan K., Lai C., Lai Z., Lin Y. (2025). Genome-wide identification and expression analysis unveil the involvement of the succinic semialdehyde dehydrogenase (*SSADH*) gene family in banana low temperature stress. Int. J. Mol. Sci..

[51] Guo Y., Bao Z., Deng Y., Li Y., Wang P. (2023). Protein subcellular localization and functional studies in horticultural research: Problems, solutions, and new approaches. Hortic. Res..

[52] Zhang Z., Kumar V., Dybkov O., Will C.L., Zhong J.Y., Ludwig S.E.J., Urlaub H., Kastner B., Stark H., Lührmann R. (2024). Structural insights into the cross-exon to cross-intron spliceosome switch. Nature.

[53] Tajo S.M., Pan Z., Jia Y., He S., Chen B., Sadau S.B., Km Y., Ajadi A.A., Nazir M.F., Auta U. (2023). Silencing of *GhORP_A02* enhances drought tolerance in *Gossypium hirsutum*. BMC Genomics.

[54] Gut H., Dominici P., Pilati S., Astegno A., Petoukhov M.V., Svergun D.I., Grütter M.G., Capitani G. (2009). A common structural basis for pH- and calmodulin-mediated regulation in plant glutamate decarboxylase. J. Mol. Biol..

[55] Shahjahan M., Rahman S. (2019). Insilico analysis of γ-aminobutyric acid transaminase (GABA-T) of *Brassica napus* (Rape). J. Adv. Biotechnol. Exp. Ther..

[56] Song X., Wang L.Y., Fu B.X., Li S.D., Wei Y.Y., Hong Y., Dai S.L. (2024). Advances in identification and synthesis of promoter elements in higher plants. Chin. J. Bot..

[57] Hong L., Liu X., Li L. (2011). Plant AREB/ABF transcription factors and their involvement in ABA signal transduction. Plant Physiol. J..

[58] Huang H., He Y., Cui A., Sun L., Han M., Wang J., Rui C., Lei Y., Liu X., Xu N. (2022). Genome-wide identification of GAD family genes suggests *GhGAD6* functionally respond to Cd^2+^ stress in cotton. Front. Genet..

[59] Wang J., Zhang Y., Wang J., Ma F., Wang L., Zhan X., Li G., Hu S., Khan A., Dang H. (2024). Promoting γ-aminobutyric acid accumulation to enhances saline-alkali tolerance in tomato. Plant Physiol..

